# Supplementary material for: Repercussions of the Calpain Cleavage-Related Missense Mutations in the Cytosolic Domains of Human Integrin-β Subunits on the Calpain–Integrin Signaling Axis
Source: Int J Mol Sci. 2025 Apr 29;26(9):4246. doi: 10.3390/ijms26094246 (PMC12071666; doi:10.3390/ijms26094246)
Supplement: Supplementary file 1 [file ijms-26-04246-s001.zip › ijms-3311024-supplementary.pdf]

Supplementary Table S1

Experimentally identified calpain substrates with cleavage sites.

Acc	Position	Code	Peptide	Pmids	calpain	Name	Gene	Organism
O00151	271	FV	LPMCDKCGTGIVGVFVKLRDRHRHPECYVC	19926129	calpain3	PDZ and LIM do	PDLIM1	*Homo sapiens*
O15527	329	AQ	QAVLFSADLRQSRHAQEPPAKRRKGSKGPE	18294929	u-calpain	N-glycosylase/DI	OGG1	*Homo sapiens*
O60216	192	LL	EDDDMLVSTTTSNLLLESEQSTSNLNEKIN	21876002	u-calpain	Double-strand-br	RAD21	*Homo sapiens*
O75084	464	LE	IRTIMKHDGTKTEKLEKLMVRIGVFSVLYT	17716656	u-calpain	Frizzled-7	FZD7	*Homo sapiens*
O75084	469	VR	KHDGTKTEKLEKLMVRIGVFSVLYTVPATI	17716656	u-calpain	Frizzled-7	FZD7	*Homo sapiens*
O94782	13	SR	**MPGVIPSESNGLSRGSPSKKNRLSLKFF	23589330	calpain	Ubiquitin carboxy	USP1	*Homo sapiens*
Q14315	2626	SS	ETVTKSSSSRGSSYSSIPKFSSDASKVVTR	16297652	u-calpain	Filamin-C	FLNC	*Homo sapiens*
P00533	683	LQ	RRRHIVRKRTLRRLLQERELVEPLTPSGEA	8055914	m-calpain	Epidermal growt	EGFR	*Homo sapiens*
P00533	733	PE	GSGAFGTVYKGLWIPEGEKVKIPVAIKELR	8055914	m-calpain	Epidermal growt	EGFR	*Homo sapiens*
P00533	1030	SR	YLIPQQGFFSSPSTSRTPLLSSLSATSNNS	8055914	m-calpain	Epidermal growt	EGFR	*Homo sapiens*
P00533	1059	PI	STVACIDRNGLQSCPIKEDSFLQRYSSDPT	8055914	m-calpain	Epidermal growt	EGFR	*Homo sapiens*
P00533	1086	FL	DPTGALTEDSIDDTFLPVPEYINQSVPKRP	8055914	m-calpain	Epidermal growt	EGFR	*Homo sapiens*
P00533	1151	FD	EYLNTVQPTCVNSTFDSPAHWAQKGSHQIS	8055914	m-calpain	Epidermal growt	EGFR	*Homo sapiens*
P00533	1185	GI	DYQQDFFPKEAKPNGIFKGSTAENAEYLRV	8055914	m-calpain	Epidermal growt	EGFR	*Homo sapiens*
P01106	298	KR	AGGHSKPPHSPLVLKRCHVSTHQHNYAAPP	20691906;212931	calpain	Myc proto-oncog	MYC	*Homo sapiens*
P01583	118	FL	SEEEIIKPRSAPFSFLSNVKYNFMRIIKYE	2115174;2201787	u-calpain	Interleukin-1 alph	IL1A	*Homo sapiens*
P02686-5	71	GS	SGKDSHHPARTAHYGSLPQKSHGRTQDENP	16412431;247238	m-calpain	Myelin basic prot	MBP	*Homo sapiens*
P02686-5	98	RT	ENPVVHFFKNIVTPRTPPPSQGKGRGLSLS	16412431;247238	m-calpain	Myelin basic prot	MBP	*Homo sapiens*
P02686-5	132	AS	GAEGQRPGFGYGGRASDYKSAHKGFKGVDA	16412431	m-calpain	Myelin basic prot	MBP	*Homo sapiens*
P02686-5	19	AS	KRPSQRHGSKYLATASTMDHARHGFLPRHR	2472383	m-calpain	Myelin basic prot	MBP	*Homo sapiens*
P02686-5	95	VT	TQDENPVVHFFKNIVTPRTPPPSQGKGRGL	2472383	m-calpain	Myelin basic prot	MBP	*Homo sapiens*
P04083	36	VS	VQTVKSSKGGPGSAVSPYPTFNPSSDVAAL	2540167	u-calpain	Annexin A1	ANXA1	*Homo sapiens*
P04275	763	RS	PDAVLSSPLSHRSKRSLSCRPPMVKLVCPA	2845608	m-calpain	von Willebrand fa	VWF	*Homo sapiens*
P04275	1913	KS	CHTVTCQPDGQTLLKSHRVNCDRGLRPSCP	2845608	m-calpain	von Willebrand fa	VWF	*Homo sapiens*
P05106	761	AR	IHDRKEFAKFEEERARAKWDTANNPLYKEA	7592818	u-calpain	Integrin beta-3	ITGB3	*Homo sapiens*
P05106	767	TA	FAKFEEERARAKWDTANNPLYKEATSTFTN	7592818;1286097	u-calpain	Integrin beta-3	ITGB3	*Homo sapiens*
P05106	773	YK	ERARAKWDTANNPLYKEATSTFTNITYRGT	7592818;1286097	u-calpain	Integrin beta-3	ITGB3	*Homo sapiens*
P05106	780	FT	DTANNPLYKEATSTFTNITYRGT*******	7592818;1286097	u-calpain	Integrin beta-3	ITGB3	*Homo sapiens*
P05106	785	YR	PLYKEATSTFTNITYRGT************	7592818;1286097	u-calpain	Integrin beta-3	ITGB3	*Homo sapiens*
P05107	724	KA	AGIVLIGILLLVIWKALIHLSDLREYRRFE	10571053	m-calpain	Integrin beta-2	ITGB2	*Homo sapiens*
P05107	744	KS	SDLREYRRFEKEKLKSQWNNDNPLFKSATT	10571053	m-calpain	Integrin beta-2	ITGB2	*Homo sapiens*
P05107	754	FK	KEKLKSQWNNDNPLFKSATTTVMNPKFAES	10571053	m-calpain	Integrin beta-2	ITGB2	*Homo sapiens*
P05107	760	TV	QWNNDNPLFKSATTTVMNPKFAES******	10571053	m-calpain	Integrin beta-2	ITGB2	*Homo sapiens*
P05107	762	MN	NNDNPLFKSATTTVMNPKFAES********	10571053	m-calpain	Integrin beta-2	ITGB2	*Homo sapiens*
P05556	771	MN	IHDRREFAKFEKEKMNAKWDTGENPIYKSA	10571053	m-calpain	Integrin beta-1	ITGB1	*Homo sapiens*
P05556	777	TG	FAKFEKEKMNAKWDTGENPIYKSAVTTVVN	10571053	m-calpain	Integrin beta-1	ITGB1	*Homo sapiens*
P05556	778	GE	AKFEKEKMNAKWDTGENPIYKSAVTTVVNP	10571053	m-calpain	Integrin beta-1	ITGB1	*Homo sapiens*
P05556	789	TV	WDTGENPIYKSAVTTVVNPKYEGK******	10571053	m-calpain	Integrin beta-1	ITGB1	*Homo sapiens*
P05556	791	VN	TGENPIYKSAVTTVVNPKYEGK********	10571053	m-calpain	Integrin beta-1	ITGB1	*Homo sapiens*
P05556-5	767	EK	LLMIIHDRREFAKFEKEKMNAKWDTQENPI	10571053	m-calpain	Integrin beta-1	ITGB1	*Homo sapiens*
P05556-5	771	MN	IHDRREFAKFEKEKMNAKWDTQENPIYKSP	10571053	m-calpain	Integrin beta-1	ITGB1	*Homo sapiens*
P05556-5	772	NA	HDRREFAKFEKEKMNAKWDTQENPIYKSPI	10571053	m-calpain	Integrin beta-1	ITGB1	*Homo sapiens*
P05556-5	777	TQ	FAKFEKEKMNAKWDTQENPIYKSPINNFKN	10571053	m-calpain	Integrin beta-1	ITGB1	*Homo sapiens*
P05556-5	778	QE	AKFEKEKMNAKWDTQENPIYKSPINNFKNP	10571053	m-calpain	Integrin beta-1	ITGB1	*Homo sapiens*
P05556-5	783	YK	EKMNAKWDTQENPIYKSPINNFKNPNYGRK	10571053	m-calpain	Integrin beta-1	ITGB1	*Homo sapiens*
P05556-5	788	NN	KWDTQENPIYKSPINNFKNPNYGRKAGL**	10571053	m-calpain	Integrin beta-1	ITGB1	*Homo sapiens*
P05556-5	789	NF	WDTQENPIYKSPINNFKNPNYGRKAGL***	10571053	m-calpain	Integrin beta-1	ITGB1	*Homo sapiens*
P05556-5	790	FK	DTQENPIYKSPINNFKNPNYGRKAGL****	10571053	m-calpain	Integrin beta-1	ITGB1	*Homo sapiens*
P05556-5	791	KN	TQENPIYKSPINNFKNPNYGRKAGL*****	10571053	m-calpain	Integrin beta-1	ITGB1	*Homo sapiens*
P05556-5	795	YG	PIYKSPINNFKNPNYGRKAGL*********	10571053	m-calpain	Integrin beta-1	ITGB1	*Homo sapiens*
P05783	17	GS	FTTRSTFSTNYRSLGSVQAPSYGARPVSSA	18408750	u-calpain	Keratin, type I cy	KRT18	*Homo sapiens*
P05783	30	SS	LGSVQAPSYGARPVSSAASVYAGAGGSGSR	18408750	u-calpain	Keratin, type I cy	KRT18	*Homo sapiens*
P05783	59	GS	RISVSRSTSFRGGMGSGGLATGIAGGLAGM	18408750	u-calpain	Keratin, type I cy	KRT18	*Homo sapiens*
P05783	64	AT	RSTSFRGGMGSGGLATGIAGGLAGMGGIQN	18408750	u-calpain	Keratin, type I cy	KRT18	*Homo sapiens*
P05783	78	QN	ATGIAGGLAGMGGIQNEKETMQSLNDRLAS	18408750	u-calpain	Keratin, type I cy	KRT18	*Homo sapiens*
P05783	80	EK	GIAGGLAGMGGIQNEKETMQSLNDRLASYL	18408750	u-calpain	Keratin, type I cy	KRT18	*Homo sapiens*
P05783	137	RA	VRDWSHYFKIIEDLRAQIFANTVDNARIVL	18408750	u-calpain	Keratin, type I cy	KRT18	*Homo sapiens*
P05783	186	RK	LAMRQSVENDIHGLRKVIDDTNITRLQLET	18408750	u-calpain	Keratin, type I cy	KRT18	*Homo sapiens*
P05783	236	EV	KGLQAQIASSGLTVEVDAPKSQDLAKIMAD	18408750	u-calpain	Keratin, type I cy	KRT18	*Homo sapiens*
P05783	253	RA	APKSQDLAKIMADIRAQYDELARKNREELD	18408750	u-calpain	Keratin, type I cy	KRT18	*Homo sapiens*
P05783	284	TQ	YWSQQIEESTTVVTTQSAEVGAAETTLTEL	18408750	u-calpain	Keratin, type I cy	KRT18	*Homo sapiens*
P05783	285	QS	WSQQIEESTTVVTTQSAEVGAAETTLTELR	18408750	u-calpain	Keratin, type I cy	KRT18	*Homo sapiens*
P05783	286	SA	SQQIEESTTVVTTQSAEVGAAETTLTELRR	18408750	u-calpain	Keratin, type I cy	KRT18	*Homo sapiens*
P05783	290	GA	EESTTVVTTQSAEVGAAETTLTELRRTVQS	18408750	u-calpain	Keratin, type I cy	KRT18	*Homo sapiens*
P05783	305	SL	AAETTLTELRRTVQSLEIDLDSMRNLKASL	18408750	u-calpain	Keratin, type I cy	KRT18	*Homo sapiens*
P05787	72	LL	SGMGGITAVTVNQSLLSPLVLEVDPNIQAV	18408750	u-calpain	Keratin, type II c	KRT8	*Homo sapiens*
P05787	73	LS	GMGGITAVTVNQSLLSPLVLEVDPNIQAVR	18408750	u-calpain	Keratin, type II c	KRT8	*Homo sapiens*
P05787	75	PL	GGITAVTVNQSLLSPLVLEVDPNIQAVRTQ	18408750	u-calpain	Keratin, type II c	KRT8	*Homo sapiens*
P05787	77	VL	ITAVTVNQSLLSPLVLEVDPNIQAVRTQEK	18408750	u-calpain	Keratin, type II c	KRT8	*Homo sapiens*
P05787	79	EV	AVTVNQSLLSPLVLEVDPNIQAVRTQEKEQ	18408750	u-calpain	Keratin, type II c	KRT8	*Homo sapiens*
P05787	236	QS	NFLRQLYEEEIRELQSQISDTSVVLSMDNS	18408750	u-calpain	Keratin, type II c	KRT8	*Homo sapiens*
P05787	440	GS	AYGGLTSPGLSYSLGSSFGSGAGSSSFSRT	18408750	u-calpain	Keratin, type II c	KRT8	*Homo sapiens*
P05787	444	GS	LTSPGLSYSLGSSFGSGAGSSSFSRTSSSR	18408750	u-calpain	Keratin, type II c	KRT8	*Homo sapiens*

P06400	810	KS	PLRIPGGNIYISPLKSPYKISEGLPTPTKM	18632617	u-calpain;m-calpain	Retinoblastoma-a	RB1	*Homo sapiens*
P07384	27	GL	GVSAQVQKQRARELGLGRHENAIKYLGQDY	1607367;2455049	u-calpain	Calpain-1 catalyt	CAPN1	*Homo sapiens*
P08581	1036	TS	RQVQYPLTDMSPILTSGDSDISSPLLQNTV	25996296	u-calpain	Hepatocyte grow	MET	*Homo sapiens*
P10415	73	QT	PAASRDPVARTSPLQTPAAPGAAAGPALSP	12000759	u-calpain;m-calpain	Apoptosis regula	BCL2	*Homo sapiens*
P11277	2058	TA	VEKLIKRHEAFEKSTASWAERFAALEKPTT	9989581	u-calpain	Spectrin beta cha	SPTB	*Homo sapiens*
P11277	2061	WA	LIKRHEAFEKSTASWAERFAALEKPTTLEL	9989581	u-calpain	Spectrin beta cha	SPTB	*Homo sapiens*
P11387	158	KK	DADYKPKKIKTEDTKKEKKRKLEEEEDGKL	21086148	m-calpain	DNA topoisomer	TOP1	*Homo sapiens*
P11387	183	KK	EDGKLKKPKNKDKDKKVPEPDNKKKKPKKE	21086148	m-calpain	DNA topoisomer	TOP1	*Homo sapiens*
P11387	207	EE	KKPKKEEEQKWKWWEEERYPEGIKWKFLEH	21086148	m-calpain	DNA topoisomer	TOP1	*Homo sapiens*
P11532	690	TR	LTQTTVMETVTTVTTREQILVKHAQEELPP	1490998	m-calpain	Dystrophin	DMD	*Homo sapiens*
P11532	1992	EI	REETMMVMTEDMPLEISYVPSTYLTEITHV	1490998	m-calpain	Dystrophin	DMD	*Homo sapiens*
P12318	255	KA	CRKKRISANSTDPVKAAQFEPPGRQMIAIR	17848620	u-calpain	Low affinity immu	FCGR2A	*Homo sapiens*
P12318	263	GR	NSTDPVKAAQFEPPGRQMIAIRKRQLEETN	17848620	u-calpain	Low affinity immu	FCGR2A	*Homo sapiens*
P12318	268	AI	VKAAQFEPPGRQMIAIRKRQLEETNNDYET	17848620	u-calpain	Low affinity immu	FCGR2A	*Homo sapiens*
P12814	243	SS	GTARPDEKAIMTYVSSFYHAFSGAQKAETA	18258589	u-calpain;m-calpain	Alpha-actinin-1	ACTN1	*Homo sapiens*
P12814	246	YH	RPDEKAIMTYVSSFYHAFSGAQKAETAANR	18258589	u-calpain;m-calpain	Alpha-actinin-1	ACTN1	*Homo sapiens*
P15311	467	MT	QDDLVKTKEELHLVMTAPPPPPPPVYEPVS	18625712	u-calpain	Ezrin	EZR	*Homo sapiens*
P16112	365	GG	GEDFVDIPENFFGVGGEEDITVQTVTWPDM	20618160	m-calpain	Aggrecan core p	ACAN	*Homo sapiens*
P16112	954	SG	AEILEGSASGVGDLSGLPSGEVLETSASGV	20618160	m-calpain	Aggrecan core p	ACAN	*Homo sapiens*
P16112	973	SG	GEVLETSASGVGDLSGLPSGEVLETTAPGV	20618160	m-calpain	Aggrecan core p	ACAN	*Homo sapiens*
P16112	1353	SG	GEVLETSTSAVGDLSGLPSGGEVLEISVSG	20618160	m-calpain	Aggrecan core p	ACAN	*Homo sapiens*
P16112	1411	SR	GEGLETSASGVEDLSRLPSGEEVLEISASG	20618160	m-calpain	Aggrecan core p	ACAN	*Homo sapiens*
P16112	1431	SG	EEVLEISASGFGDLSGVPSGGEGLETSASE	20618160	m-calpain	Aggrecan core p	ACAN	*Homo sapiens*
P16112	1452	SG	EGLETSASEVGTDLSGLPSGREGLETSASG	20618160	m-calpain	Aggrecan core p	ACAN	*Homo sapiens*
P16112	1472	SG	REGLETSASGAEDLSGLPSGKEDLVGSASG	20618160	m-calpain	Aggrecan core p	ACAN	*Homo sapiens*
P16112	1573	GA	TASELEGRGTIGISGAGEISGLPSSELDIS	20618160	m-calpain	Aggrecan core p	ACAN	*Homo sapiens*
P16112	1681	SG	ITDLSGETSGVPDLSGQPSGLPGFSGATSG	20618160	m-calpain	Aggrecan core p	ACAN	*Homo sapiens*
P16112	709	AA	GVEEWIVTQVVPGVAAVPVEEETTAVPSGE	20618160;172615	m-calpain	Aggrecan core p	ACAN	*Homo sapiens*
P17252	309	KL	EEGNMELRQKFEKAKLGPAGNKVISPSEDR	2537303	u-calpain	Protein kinase C	PRKCA	*Homo sapiens*
P17252	316	KV	RQKFEKAKLGPAGNKVISPSEDRKQPSNNL	20689063;253730	u-calpain	Protein kinase C	PRKCA	*Homo sapiens*
P17252	324	RK	LGPAGNKVISPSEDRKQPSNNLDRVKLTDF	20689063;253730	u-calpain	Protein kinase C	PRKCA	*Homo sapiens*
P17936	27	AG	LTLLVLLRGPPVARAGASSAGLGPVVRCEP	17655506	m-calpain	Insulin-like growt	IGFBP3	*Homo sapiens*
P17936	169	HS	SSTHRVSDPKFHPLHSKIIIIKKGHAKDSQ	17655506	m-calpain	Insulin-like growt	IGFBP3	*Homo sapiens*
P18065	36	AE	LLLLGASGGGGGARAEVLFRCPPCTPERLA	17655506	m-calpain	Insulin-like growt	IGFBP2	*Homo sapiens*
P18065	199	HR	MKELAVFREKVTEQHRQMGKGGKHHLGLEE	17655506;245504	m-calpain	Insulin-like growt	IGFBP2	*Homo sapiens*
P21333	1761	YT	SVQPPLRSQQLAPQYTYAQGGQQTWAPERP	22870205;149883	calpain	Filamin-A	FLNA	*Homo sapiens*
P22692	23	EA	ALLLAAGPGPSLGDEAIHCPPCSEEKLARC	15843151	u-calpain	Insulin-like growt	IGFBP4	*Homo sapiens*
P22692	107	EA	LMHGQGVCMELAEIEAIQESLQPSDKDEGD	15843151	u-calpain	Insulin-like growt	IGFBP4	*Homo sapiens*
P22692	143	FA	SPCSAHDRRCLQKHFAKIRDRSTSGGKMKV	15843151	u-calpain	Insulin-like growt	IGFBP4	*Homo sapiens*
P22692	159	NG	KIRDRSTSGGKMKVNGAPREDARPVPQGSC	15843151	u-calpain	Insulin-like growt	IGFBP4	*Homo sapiens*
P23109	85	SI	RRKKRFQGRKTVNLSIPLSETSSTKLSHID	9857047;1548877	m-calpain	AMP deaminase	AMPD1	*Homo sapiens*
P23109	97	SH	NLSIPLSETSSTKLSHIDEYISSSPTYQTV	9857047;1548877	m-calpain	AMP deaminase	AMPD1	*Homo sapiens*
P24593	22	GS	LLLLAAYAGPAQSLGSFVHCEPCDEKALSM	15843151	u-calpain	Insulin-like growt	IGFBP5	*Homo sapiens*
P24593	161	TQ	LKAEAVKKDRRKKLTQSKFVGGAENTAHPR	15843151	u-calpain	Insulin-like growt	IGFBP5	*Homo sapiens*
P24593	172	TA	KKLTQSKFVGGAENTAHPRIISAPEMRQES	15843151	u-calpain	Insulin-like growt	IGFBP5	*Homo sapiens*
P25963	50	QE	SMKDEEYEQMVKELQEIRLEPQEVPRGSEP	15202778;245504	m-calpain	NF-kappa-B inhil	NFKBIA	*Homo sapiens*
P26010	746	YR	VGGIVAVGLGLVLAYRLSVEIYDRREYSRF	10571053	m-calpain	Integrin beta-7	ITGB7	*Homo sapiens*
P26010	760	RF	YRLSVEIYDRREYSRFEKEQQQLNWKQDSN	10571053	m-calpain	Integrin beta-7	ITGB7	*Homo sapiens*
P26010	765	QQ	EIYDRREYSRFEKEQQQLNWKQDSNPLYKS	10571053	m-calpain	Integrin beta-7	ITGB7	*Homo sapiens*
P26010	766	QQ	IYDRREYSRFEKEQQQLNWKQDSNPLYKSA	10571053	m-calpain	Integrin beta-7	ITGB7	*Homo sapiens*
P26010	769	NW	RREYSRFEKEQQQLNWKQDSNPLYKSAITT	10571053	m-calpain	Integrin beta-7	ITGB7	*Homo sapiens*
P26010	770	WK	REYSRFEKEQQQLNWKQDSNPLYKSAITTT	10571053	m-calpain	Integrin beta-7	ITGB7	*Homo sapiens*
P26010	773	DS	SRFEKEQQQLNWKQDSNPLYKSAITTTINP	10571053	m-calpain	Integrin beta-7	ITGB7	*Homo sapiens*
P26010	774	SN	RFEKEQQQLNWKQDSNPLYKSAITTTINPR	10571053	m-calpain	Integrin beta-7	ITGB7	*Homo sapiens*
P26010	778	YK	EQQQLNWKQDSNPLYKSAITTTINPRFQEA	10571053	m-calpain	Integrin beta-7	ITGB7	*Homo sapiens*
P26010	784	TT	WKQDSNPLYKSAITTTINPRFQEADSPTL*	10571053	m-calpain	Integrin beta-7	ITGB7	*Homo sapiens*
P26010	785	TI	KQDSNPLYKSAITTTINPRFQEADSPTL**	10571053	m-calpain	Integrin beta-7	ITGB7	*Homo sapiens*
P26599	163	AL	AALQAVNSVQSGNLALAASAAAVDAGMAMA	18408750	u-calpain	Polypyrimidine tr	PTBP1	*Homo sapiens*
P26599	165	AA	LQAVNSVQSGNLALAASAAAVDAGMAMAGQ	18408750	u-calpain	Polypyrimidine tr	PTBP1	*Homo sapiens*
P30301	237	LK	LFPRLKSISERLSVLKGAKPDVSNGQPEVT	16310784	m-calpain	Lens fiber major	MIP	*Homo sapiens*
P30301	238	KG	FPRLKSISERLSVLKGAKPDVSNGQPEVTG	16310784;100679	m-calpain	Lens fiber major	MIP	*Homo sapiens*
P30301	252	TG	KGAKPDVSNGQPEVTGEPVELNTQAL****	16310784	m-calpain	Lens fiber major	MIP	*Homo sapiens*
P30301	259	NT	SNGQPEVTGEPVELNTQAL***********	16310784;160541	m-calpain	Lens fiber major	MIP	*Homo sapiens*
P30301	260	TQ	NGQPEVTGEPVELNTQAL************	16310784	m-calpain	Lens fiber major	MIP	*Homo sapiens*
P30301	239	GA	PRLKSISERLSVLKGAKPDVSNGQPEVTGE	16054132	m-calpain	Lens fiber major	MIP	*Homo sapiens*
P30301	246	NG	ERLSVLKGAKPDVSNGQPEVTGEPVELNTQ	16054132	m-calpain	Lens fiber major	MIP	*Homo sapiens*
P31944	152	IK	DPGETVGGDEIVMVIKDSPQTIPTYTDALH	12200134;153804	u-calpain	Caspase-14	CASP14	*Homo sapiens*
P32119	181	DT	EHGEVCPAGWKPGSDTIKPNVDDSKEYFSK	9602152	u-calpain	Peroxiredoxin-2	PRDX2	*Homo sapiens*
P32119	182	TI	HGEVCPAGWKPGSDTIKPNVDDSKEYFSKH	9602152	u-calpain;m-calpain	Peroxiredoxin-2	PRDX2	*Homo sapiens*
P32119	183	IK	GEVCPAGWKPGSDTIKPNVDDSKEYFSKHN	9602152	u-calpain;m-calpain	Peroxiredoxin-2	PRDX2	*Homo sapiens*
P32119	193	YF	GSDTIKPNVDDSKEYFSKHN**********	9602152	u-calpain;m-calpain	Peroxiredoxin-2	PRDX2	*Homo sapiens*
P32119	194	FS	SDTIKPNVDDSKEYFSKHN***********	9602152	u-calpain;m-calpain	Peroxiredoxin-2	PRDX2	*Homo sapiens*
P33316-2	4	SE	***********MPCSEETPAISPSKRARPA	21625588	m-calpain	Sodium- and chlo	Slc6a9	*Homo sapiens*
P33316-2	7	TP	********MPCSEETPAISPSKRARPAEVG	21625588	m-calpain	Sodium- and chlo	Slc6a9	*Homo sapiens*
P33316-2	31	LS	RPAEVGGMQLRFARLSEHATAPTRGSARAA	21625588	m-calpain	Sodium- and chlo	Slc6a9	*Homo sapiens*
P35240	294	KL	IDVFKFNSSKLRVNKLILQLCIGNHDLFMR	9701243	u-calpain;m-calpain	Merlin	NF2	*Homo sapiens*
P35240	298	QL	KFNSSKLRVNKLILQLCIGNHDLFMRRRKA	9701243	u-calpain;m-calpain	Merlin	NF2	*Homo sapiens*

P37840	73	GV	TKEQVTNVGGAVVTGVTAVAQKTVEGAGSI	12887682	u-calpain	Alpha-synuclein	SNCA	*Homo sapiens*
P37840	75	TA	EQVTNVGGAVVTGVTAVAQKTVEGAGSIAA	12887682	u-calpain	Alpha-synuclein	SNCA	*Homo sapiens*
P37840	83	EG	AVVTGVTAVAQKTVEGAGSIAAATGFVKKD	12887682	u-calpain	Alpha-synuclein	SNCA	*Homo sapiens*
P37840	18	AA	FMKGLSKAKEGVVAAAEKTKQGVAEAAGKT	17005155	m-calpain	Alpha-synuclein	SNCA	*Homo sapiens*
P37840	31	GK	AAAEKTKQGVAEAAGKTKEGVLYVGSKTKE	17005155	m-calpain	Alpha-synuclein	SNCA	*Homo sapiens*
P37840	39	YV	GVAEAAGKTKEGVLYVGSKTKEGVVHGVAT	17005155	m-calpain	Alpha-synuclein	SNCA	*Homo sapiens*
P37840	57	EK	KTKEGVVHGVATVAEKTKEQVTNVGGAVVT	12887682;170051	u-calpain;m-calpain	Alpha-synuclein	SNCA	*Homo sapiens*
P37840	114	ED	LGKNEEGAPQEGILEDMPVDPDNEAYEMPS	12887682;250092	u-calpain	Alpha-synuclein	SNCA	*Homo sapiens*
P37840	122	NE	PQEGILEDMPVDPDNEAYEMPSEEGYQDYE	12887682;250092	u-calpain	Alpha-synuclein	SNCA	*Homo sapiens*
P42858	467	TA	DDSESRSDVSSSALTASVKDEISGELAASS	14981075	u-calpain;m-calpain	Huntingtin	HTT	*Homo sapiens*
P42858	534	SS	SATDGDEEDILSHSSSQVSAVPSDPAMDLN	14981075	u-calpain;m-calpain	Huntingtin	HTT	*Homo sapiens*
P47897	198	ET	DLEKKFKVAKARLEETDRRTAKDVVENGET	26324710	m-calpain	Glutamine--tRNA	QARS	*Homo sapiens*
P49418	333	SV	NIISFFEDNFVPEISVTTPSQNEVPEVKKE	17541403	m-calpain	Amphiphysin	AMPH	*Homo sapiens*
P49418	377	SQ	VTPAGSAGVTHSPMSQTLPWDLWTTSTDLV	17541403	m-calpain	Amphiphysin	AMPH	*Homo sapiens*
P49418	392	VQ	QTLPWDLWTTSTDLVQPASGGSFNGFTQPQ	17541403	m-calpain	Amphiphysin	AMPH	*Homo sapiens*
P49418	454	MD	PLAAVTPAVGLDLGMDTRAEEPVEEAVIIP	17541403	m-calpain	Amphiphysin	AMPH	*Homo sapiens*
P49418	478	GT	EAVIIPGADADAAVGTLVSAAEGAPGEEAE	17541403	m-calpain	Amphiphysin	AMPH	*Homo sapiens*
P49418	527	AE	GTETTEGAESAQPEAEELEATVPQEKVIPS	17541403	m-calpain	Amphiphysin	AMPH	*Homo sapiens*
P49418	531	EA	TEGAESAQPEAEELEATVPQEKVIPSVVIE	17541403	m-calpain	Amphiphysin	AMPH	*Homo sapiens*
P49418	593	DP	SETPELATEQKPIQDPQPTPSAPAMGAADQ	17541403	m-calpain	Amphiphysin	AMPH	*Homo sapiens*
P49418	609	LA	QPTPSAPAMGAADQLASAREASQELPPGFL	17541403	m-calpain	Amphiphysin	AMPH	*Homo sapiens*
P53618	528	SS	LVTEMGTYATQSALSSSRPTKKEEDRPPLR	16476741	u-calpain	Coatomer subun	COPB1	*Homo sapiens*
P53805-2	6	FN	*********MHFRNFNYSFSSLIACVANSD	15935327	u-calpain	Calcipressin-1	RCAN1	*Homo sapiens*
P53805-2	133	YA	QVEDATPVINYDLLYAISKLGPGEKYELHA	15935327	u-calpain	Calcipressin-1	RCAN1	*Homo sapiens*
P54136	63	QA	LKLKYRLNILRKSLQAERNKPTKNMINIIS	26324710	u-calpain	Arginine--tRNA li	RARS	*Homo sapiens*
P54252	55	SE	DEEERMRMAEGGVTSEDYRTFLQQPSGNMD	17488727	m-calpain	Ataxin-3	ATXN3	*Homo sapiens*
P54252	62	LQ	MAEGGVTSEDYRTFLQQPSGNMDDSGFFSI	17488727	m-calpain	Ataxin-3	ATXN3	*Homo sapiens*
P54252	187	HR	EADQLLQMIRVQQMHRPKLIGEELAQLKEQ	17488727	m-calpain	Ataxin-3	ATXN3	*Homo sapiens*
P54252	208	DL	EELAQLKEQRVHKTDLERVLEANDGSGMLD	17488727	m-calpain	Ataxin-3	ATXN3	*Homo sapiens*
P54252	256	SM	MEDEEADLRRAIQLSMQGSSRNISQDMTQT	17488727	m-calpain	Ataxin-3	ATXN3	*Homo sapiens*
P54252	305	QG	KQQQKQQQQQQQQQGDLSGQSSHPCERPA	17488727	m-calpain	Ataxin-3	ATXN3	*Homo sapiens*
P54252	260	SS	EADLRRAIQLSMQGSSRNISQDMTQTSGTN	23100324	m-calpain	Ataxin-3	ATXN3	*Homo sapiens*
P55210	36	FS	VDAKPDRSSFVPSLFSKKKKNVTMRSIKTT	19617626	u-calpain;m-calpain	Caspase-7	CASP7	*Homo sapiens*
P55210	45	MR	FVPSLFSKKKKNVTMRSIKTTRDRVPTYQY	19617626	u-calpain;m-calpain	Caspase-7	CASP7	*Homo sapiens*
P55210	47	SI	PSLFSKKKKNVTMRSIKTTRDRVPTYQYNM	19617626	u-calpain;m-calpain	Caspase-7	CASP7	*Homo sapiens*
P55211	115	IR	PTLENLTPVVLRPEIRKPEVLRPETPRPVD	10671558	m-calpain	Caspase-9	CASP9	*Homo sapiens*
P55211	330	DA	ATPFQEGLRTFDQLDAISSLPTPSDIFVSY	10671558	m-calpain	Caspase-9	CASP9	*Homo sapiens*
P55273	25	RR	RLSGAAARGDVQEVRRLLHRELVHPDALNR	16542156	u-calpain	Cyclin-dependen	CDKN2D	*Homo sapiens*
P55273	29	HR	AAARGDVQEVRRLLHRELVHPDALNRFGKT	16542156	u-calpain	Cyclin-dependen	CDKN2D	*Homo sapiens*
P55273	47	QV	VHPDALNRFGKTALQVMMFGSTAIALELLK	16542156	u-calpain	Cyclin-dependen	CDKN2D	*Homo sapiens*
P55273	64	GA	MFGSTAIALELLKQGASPNVQDTSGTSPVH	16542156	u-calpain	Cyclin-dependen	CDKN2D	*Homo sapiens*
P55273	113	LA	DVNVPDGTGALPIHLAVQEGHTAVVSFLAA	16542156	u-calpain	Cyclin-dependen	CDKN2D	*Homo sapiens*
P55273	127	AA	LAVQEGHTAVVSFLAAESDLHRRDARGLTP	16542156	u-calpain	Cyclin-dependen	CDKN2D	*Homo sapiens*
P55957	54	YD	LGHELPVLAPQWEGYDELQTDGNRSSHSRL	11404357;120007	u-calpain;m-calpain	BH3-interacting	BID	*Homo sapiens*
P55957	70	GR	ELQTDGNRSSHSRLGRIEADSESQEDIIRN	11940658;250569	m-calpain	BH3-interacting	BID	*Homo sapiens*
P57103	370	KK	IQATRMMTGAGNILKKHAAEQAKKASSMSE	15680332;228558	u-calpain;m-calpain	Sodium/calcium	SLC8A3	*Homo sapiens*
P57103	504	NS	EEEQPEEGMPPAIFNSLPLPRAVLASPCVA	15680332;228558	u-calpain;m-calpain	Sodium/calcium	SLC8A3	*Homo sapiens*
P57103	510	RA	EGMPPAIFNSLPLPRAVLASPCVATVTILD	15680332;228558	u-calpain;m-calpain	Sodium/calcium	SLC8A3	*Homo sapiens*
P57103	512	VL	MPPAIFNSLPLPRAVLASPCVATVTILDDD	15680332;228558	u-calpain;m-calpain	Sodium/calcium	SLC8A3	*Homo sapiens*
P60709	37	RP	GDDAPRAVFPSIVGRPRHQGVMVGMGQKDS	9472000	calpain	Actin, cytoplasmi	ACTB	*Homo sapiens*
P61586	180	QA	GVREVFEMATRAALQARRGKKKSGCLVL**	11964413	u-calpain	Transforming pr	RHOA	*Homo sapiens*
P62736	39	RP	GDDAPRAVFPSIVGRPRHQGVMVGMGQKDS	9472000	calpain	Actin, aortic smo	ACTA2	*Homo sapiens*
P68871	9	KS	******MVHLTPEEKSAVTALWGKVNVDEV	6331510	u-calpain	Hemoglobin subu	HBB	*Homo sapiens*
P69905	12	KA	***MVLSPADKTNVKAAWGKVGAHAGEYGA	6331510	u-calpain	Hemoglobin subu	HBA1	*Homo sapiens*
P78318	255	FG	KPFILTRNMAQAKVFGAGYPSLPTMTVSDW	22613722	calpain	Immunoglobulin-	IGBP1	*Homo sapiens*
Q01082	2066	AA	VEKLIKRHEAFEKSAATWDERFSALERLTT	9989581	u-calpain	Spectrin beta cha	SPTBN1	*Homo sapiens*
Q01082	1440	QS	ENQMEVRKKEIEELQSQAQALSQEGKSTDE	17209560	u-calpain	Spectrin beta cha	SPTBN1	*Homo sapiens*
Q01082	1447	SQ	KKEIEELQSQAQALSQEGKSTDEVDSKRLT	17209560	u-calpain	Spectrin beta cha	SPTBN1	*Homo sapiens*
Q01082	1467	FM	TDEVDSKRLTVQTKFMELLEPLNERKHNLL	17209560	u-calpain	Spectrin beta cha	SPTBN1	*Homo sapiens*
Q01082	1482	LA	MELLEPLNERKHNLLASKEIHQFNRDVEDE	17209560	u-calpain	Spectrin beta cha	SPTBN1	*Homo sapiens*
Q01432	87	SL	KRKKSFKMIRSQSLSLQMPPQQDWKGPPAA	9857047;1548877	m-calpain	AMP deaminase	AMPD3	*Homo sapiens*
Q01432	89	QM	KKSFKMIRSQSLSLQMPPQQDWKGPPAASP	9857047;1548877	m-calpain	AMP deaminase	AMPD3	*Homo sapiens*
Q01814	1124	RR	NEDVEEIDHAERELRRGQILWFRGLNRIQT	2542272	u-calpain	Plasma membra	ATP2B2	*Homo sapiens*
Q01814	1135	NR	RELRRGQILWFRGLNRIQTQIRVVKAFRSS	2542272	u-calpain	Plasma membra	ATP2B2	*Homo sapiens*
Q01814	1144	VK	WFRGLNRIQTQIRVVKAFRSSLYEGLEKPE	2542273	u-calpain	Plasma membra	ATP2B3	*Homo sapiens*
Q01959	43	TS	VELILVKEQNGVQLTSSTLTNPRQSPVEAQ	18468730	m-calpain	Sodium-depende	SLC6A3	*Homo sapiens*
Q01959	71	LS	AQDRETWGKKIDFLLSVIGFAVDLANVWRF	18468730	m-calpain	Sodium-depende	SLC6A3	*Homo sapiens*
Q05329	69	RK	PAESGGSQPPRAAARKAACACDQKPCSCSK	14576464	calpain	Glutamate decar	GAD2	*Homo sapiens*
Q05397	745	SG	PSPQHMVQTNHYQVSGYPGSHGITAMAGSI	20150423	m-calpain	Focal adhesion k	PTK2	*Homo sapiens*
Q07812	28	QG	TSSEQIMKTGALLLQGFIQDRAGRMGGEAP	9764817;1249031	calpain	Apoptosis regula	BAX	*Homo sapiens*
Q07817	42	ES	SDVEENRTEAPEGTESEMETPSAINGNPSW	12000759	u-calpain;m-calpain	Bcl-2-like protein	BCL2L1	*Homo sapiens*
Q07817	60	AD	ETPSAINGNPSWHLADSPAVNGATGHSSSL	10953012;245504	m-calpain	Bcl-2-like protein	BCL2L1	*Homo sapiens*
Q07820	162	ST	GESGNNTSTDGSLPSTPPPAEEEEDELYRQ	20392693	u-calpain	Induced myeloid	MCL1	*Homo sapiens*
Q08209	441	KQ	TPTGMLPSGVLSGGKQTLQSATVEAIEADE	20107181	m-calpain	Serine/threonine-	PPP3CA	*Homo sapiens*
Q08209	392	RK	GSEEDGFDGATAAARKEVIRNKIRAIGKMA	14627704	m-calpain	Serine/threonine-	PPP3CA	*Homo sapiens*
Q08209	424	KG	FSVLREESESVLTLKGLTPTGMLPSGVLSG	14627704;157239	m-calpain	Serine/threonine-	PPP3CA	*Homo sapiens*
Q08209	501	KA	RDAMPSDANLNSINKALTSETNGTDSNGSN	14627704;161506	u-calpain;m-calpain	Serine/threonine-	PPP3CA	*Homo sapiens*

Q12904	106	SS	VSENVIQSTAVTTVSSGTKEQIKGGTGDEK	26324710	m-calpain	Aminoacyl tRNA	AIMP1	*Homo sapiens*
Q13255	936	SY	TACNQTAVIKPLTKSYQGSGKSLTFSDTST	17270736;245504	u-calpain	Metabotropic glu	GRM1	*Homo sapiens*
Q13813	1176	YG	LMAEEVQAVQQQEVYGMMPRDETDSKTASP	2844821;2850618	u-calpain;m-calpain	Spectrin alpha ch	SPTAN1	*Homo sapiens*
Q13813	1230	GS	RSLQQLAEERSQLLGSAHEVQRFHRDADET	2844821;2850618	u-calpain;m-calpain	Spectrin alpha ch	SPTAN1	*Homo sapiens*
Q14315	2626	SS	ETVTKSSSSRGSSYSSIPKFSSDASKVVTR	16297652	u-calpain	Filamin-C	FLNC	*Homo sapiens*
Q14814	78	SR	KVLLKYTEYNEPHESRTNADIIETLRKKGF	22215669	m-calpain	Myocyte-specific	MEF2D	*Homo sapiens*
Q16620	521	NS	KIPVIENPQYFGITNSQLKPDTFVQHIKRH	24860020	m-calpain	BDNF/NT-3 grov	NTRK2	*Homo sapiens*
Q16637	192	SF	NKSDNIKPKSAPWNSFLPPPPPMPGPRLGP	21209906	u-calpain	Survival motor ne	SMN1	*Homo sapiens*
Q16637	193	FL	KSDNIKPKSAPWNSFLPPPPPMPGPRLGPG	21209906	u-calpain	Survival motor ne	SMN1	*Homo sapiens*
Q16849	608	DK	LAVALCVRQHARQQDKERLAALGPEGAHGD	11483505;245504	u-calpain	Receptor-type ty	PTPRN	*Homo sapiens*
Q16849	658	SS	RAEGPPEPSRVSSVSSQFSDAAQASPSSHS	11483505	u-calpain	Receptor-type ty	PTPRN	*Homo sapiens*
Q5D862	1332	ST	RGDTTRHGHSGHGQSTQTGSRTSGRQRFSH	21531719	u-calpain	Filaggrin-2	FLG2	*Homo sapiens*
Q5D862	1469	HG	GESGSTVHGRHGTTHGQTGDTTRHAHYHHG	21531719	u-calpain	Filaggrin-2	FLG2	*Homo sapiens*
Q5D862	1713	TT	TGDTTRHAHYHHGLTTQTGSRTTGRRGSGH	21531719	u-calpain	Filaggrin-2	FLG2	*Homo sapiens*
Q5D862	1741	SH	GHSEYSDSEGYSGVSHTHSGHTHGQARSQH	21531719	u-calpain	Filaggrin-2	FLG2	*Homo sapiens*
Q6NZI2	30	QA	YPDAEAPEPSSAGAQAAEEPSGAGSEELIK	15242332	calpain	Polymerase I and	PTRF	*Homo sapiens*
Q6NZI2	370	HA	GEAGDLRRGSSPDVHALLEITEESDAVLVD	15242332	calpain	Polymerase I and	PTRF	*Homo sapiens*
Q86UX7	373	YR	FRIPRRPRKLTLKGYRQHWVVFKETTLSYY	23012377	u-calpain	Fermitin family h	FERMT3	*Homo sapiens*
Q8NE35	441	RK	PTRCQNGERVERYSRKVFVGGLPPDIDEDE	22711986	m-calpain	Cytoplasmic poly	CPEB3	*Homo sapiens*
Q99259	70	RQ	SLEEKSRLVSAFKERQSSKNLLSCENSDRD	18599042;185990	u-calpain;m-calpain	Glutamate decar	GAD1	*Homo sapiens*
Q99259	90	RT	LLSCENSDRDARFRRTETDFSNLFARDLLP	18599042;185990	u-calpain;m-calpain	Glutamate decar	GAD1	*Homo sapiens*
Q9H1Y0	193	TT	ENGFRYIPFRIYQTTTERPFIQKLFRPVAA	16998475	u-calpain;m-calpain	Autophagy protei	ATG5	*Homo sapiens*
Q9ULH0	1677	NR	PEENWPACQKAYNLNRTPSTVTLNNNSAPA	26492372	u-calpain	Kinase D-interac	KIDINS220	*Homo sapiens*
Q9Y490	2493	KM	AFEEQENETVVVKEKMVGGIAQIIAAQEEM	22496808	m-calpain	Talin-1	TLN1	*Homo sapiens*
Q9Y490	433	QQ	MLEDSVSPKKSTVLQQQYNRVGKVEHGSVA	10545199;212059	m-calpain	Talin-1	TLN1	*Homo sapiens*

Supplementary Table S2

gene_symbol	gene_id	mutation_subtype	genomic_DNA_change	calpain	Activities / Pathway involvement	Reference for activities/PW
PPP3C	ENSG00000138814	Single base substitution	chr4:g.101099614C>T	m-calpain; u-calpain	MAPK signaling P/W Calcium signaling P/W	https://www.kegg.jp/ kegg-
KIDIN	ENSG00000134313	Single base substitution	chr2:g.8817625C>T	u-calpain	Neurotrophin Signaling P/w	https://www.kegg.jp/ kegg- bin/search_pathway_ text?map=hsa&keyw
KIDIN	ENSG00000134313	Single base substitution	chr2:g.8793957G>A	u-calpain		
KIDIN	ENSG00000134313	Single base substitution	chr2:g.8770674C>T	u-calpain		
KIDIN	ENSG00000134313	Single base substitution	chr2:g.8750290T>C	u-calpain		
DMD	ENSG00000198947	Single base substitution	chrX:g.31679535C>T	m-calpain	Cardiomyopathy P/Ws Salmonella infection NOD like receptor P/W Cytosolic DNA sensing P/W	https://www.kegg.jp/ kegg- bin/search_pathway_ text?map=hsa&keyw ord=DMD&mode=1 &viewImage=true
DMD	ENSG00000198947	Single base substitution	chrX:g.32438341C>T	m-calpain		
DMD	ENSG00000198947	Single base substitution	chrX:g.31478192G>A	m-calpain		
DMD	ENSG00000198947	Single base substitution	chrX:g.31178720C>T	m-calpain		
DMD	ENSG00000198947	Single base substitution	chrX:g.32448602C>A	m-calpain		
DMD	ENSG00000198947	Single base substitution	chrX:g.32454769G>T	m-calpain		
DMD	ENSG00000198947	Single base substitution	chrX:g.32501818T>C	m-calpain		
DMD	ENSG00000198947	Single base substitution	chrX:g.31206662C>T	m-calpain		
DMD	ENSG00000198947	Single base substitution	chrX:g.32310147G>T	m-calpain		
DMD	ENSG00000198947	Single base substitution	chrX:g.32386380G>A	m-calpain		
DMD	ENSG00000198947	Single base substitution	chrX:g.32816552C>T	m-calpain		
DMD	ENSG00000198947	Single base substitution	chrX:g.31169530C>T	m-calpain		
DMD	ENSG00000198947	Single base substitution	chrX:g.31478156C>T	m-calpain		
DMD	ENSG00000198947	Single base substitution	chrX:g.31223062G>T	m-calpain		
SLC6A	ENSG00000196517	Single base substitution	chr1:g.44002936G>A	m-calpain	Synaptic vesicle cycle	https://www.kegg.jp/ kegg-
SLC6A	ENSG00000196517	Single base substitution	chr1:g.44002592G>A	m-calpain		
SPTAN	ENSG00000197694	Single base substitution	chr9:g.128583884C>T	u-calpain; m-calpain	Apoptosis	https://www.kegg.jp/ kegg- bin/search_pathway_ text?map=hsa&keyw ord=SPTAN1&mod e=1&viewImage=tru e
SPTAN	ENSG00000197694	Single base substitution	chr9:g.128587654G>A	u-calpain; m-calpain		
SPTAN	ENSG00000197694	Single base substitution	chr9:g.128582737G>A	u-calpain; m-calpain		
SPTAN	ENSG00000197694	Single base substitution	chr9:g.128621212C>T	u-calpain; m-calpain		
PTPRN	ENSG00000054356	Single base substitution	chr2:g.219290837C>T	u-calpain	Type I diabetes mellitus	https://www.kegg.jp/ kegg- bin/search_pathway_
PTPRN	ENSG00000054356	Single base substitution	chr2:g.219299358C>T	u-calpain		
PTPRN	ENSG00000054356	Single base substitution	chr2:g.219290853C>T	u-calpain		
ITGB2	ENSG00000160255	Single base substitution	chr21:g.44899098G>A	m-calpain	Cell adhesion molecules, Leukocyte transendothelial	https://www.kegg.jp/ kegg- bin/search_pathway_
ITGB2	ENSG00000160255	Single base substitution	chr21:g.44900448G>A	m-calpain		
ITGB2	ENSG00000160255	Single base substitution	chr21:g.44890008G>A	m-calpain		
FLNA	ENSG00000196924	Single base substitution	chrX:g.154362501G>A	calpain	Proteoglycans in cancer, MAPK signaling P/W, Focal adhesion	https://www.kegg.jp/ kegg- bin/search_pathway_ text?map=hsa&keyw
FLNA	ENSG00000196924	Single base substitution	chrX:g.154354217G>A	calpain		
FLNA	ENSG00000196924	Single base substitution	chrX:g.154366126G>A	calpain		
FLNA	ENSG00000196924	Single base substitution	chrX:g.154354220C>T	calpain		
ITGB1	ENSG00000150093	Single base substitution	chr10:g.32908369G>A	m calpain	ECM receptor interaction, Proteoglycans in cancer, Cell adhesion molecules, Leukocyte transendothelial migration, Rap1 signaling pathway, Focal adhesion, PI3K-Akt signaling pathway	https://www.kegg.jp/ kegg- bin/search_pathway_ text?map=hsa&keyw ord=ITGB1&mode= 1&viewImage=true
NF2	ENSG00000186575	Single base substitution	chr22:g.29673398C>T	u-calpain ; m-calpain	Tight junction	https://www.kegg.jp/ kegg- bin/search_pathway_ text?map=hsa&keyw
NF2	ENSG00000186575	Single base substitution	chr22:g.29665050C>T	u-calpain ; m-calpain		

COPB	ENSG00000129083	Single base substitution	chr11:g.14486465G>A	u-calpain	Nil	
COPB	ENSG00000129083	Single base substitution	chr11:g.14483079G>A	u-calpain		
COPB	ENSG00000129083	Single base substitution	chr11:g.14461221G>A	u-calpain		
COPB	ENSG00000129083	Single base substitution	chr11:g.14479654G>A	u-calpain		
COPB	ENSG00000129083	Single base substitution	chr11:g.14458546G>A	u-calpain		
COPB	ENSG00000129083	Single base substitution	chr11:g.14493693C>T	u-calpain		
COPB	ENSG00000129083	Single base substitution	chr11:g.14461269C>T	u-calpain		
COPB	ENSG00000129083	Single base substitution	chr11:g.14493712G>A	u-calpain		
COPB	ENSG00000129083	Single base substitution	chr11:g.14493769G>A	u-calpain		
FLNC	ENSG00000128591	Single base substitution	chr7:g.128846391G>A	u-calpain	Proteoglycans in cancer, MAPK signaling P/W, Focal adhesion	https://www.kegg.jp/kegg-bin/search_pathway_text?map=hsa&keyword=FLNC&mode=1&viewImage=true
FLNC	ENSG00000128591	Single base substitution	chr7:g.128853013G>A	u-calpain		
FLNC	ENSG00000128591	Single base substitution	chr7:g.128838740G>A	u-calpain		
FLNC	ENSG00000128591	Single base substitution	chr7:g.128852944G>A	u-calpain		
FLNC	ENSG00000128591	Single base substitution	chr7:g.128848607C>T	u-calpain		
FLNC	ENSG00000128591	Single base substitution	chr7:g.128842862G>A	u-calpain		
FLNC	ENSG00000128591	Single base substitution	chr7:g.128855218C>G	u-calpain		
FLNC	ENSG00000128591	Single base substitution	chr7:g.128840671G>A	u-calpain		
FLNC	ENSG00000128591	Single base substitution	chr7:g.128847711G>A	u-calpain		
FLNC	ENSG00000128591	Single base substitution	chr7:g.128853795G>A	u-calpain		
FLNC	ENSG00000128591	Single base substitution	chr7:g.128842883G>A	u-calpain		
ITGB3	ENSG00000259207	Single base substitution	chr17:g.47310194G>A	u-calpain	ECM-receptor interaction, MicroRNAs in cancer, Rap1 signaling pathway, PI3K-Akt	https://www.kegg.jp/kegg-bin/search_pathway_text?map=hsa&keyw
ITGB3	ENSG00000259207	Single base substitution	chr17:g.47307581G>A	u-calpain		
ITGB3	ENSG00000259207	Single base substitution	chr17:g.47290960C>T	u-calpain		
ITGB3	ENSG00000259207	Single base substitution	chr17:g.47284607C>T	u-calpain		
EGFR	ENSG00000146648	Single base substitution	chr7:g.55191743C>T	m-calpain	EGFR tyrosine kinase inhibitor resistance, VEGF signaling pathway, Non-small cell lung cancer, ErbB signaling pathway, Breast cancer, Glioma	https://www.kegg.jp/kegg-bin/search_pathway_text?mode=2&viewImage=true&keyword=EGFR&map=hsa&uniqid=2023081419
EGFR	ENSG00000146648	Single base substitution	chr7:g.55146623G>A	m-calpain		
EGFR	ENSG00000146648	Single base substitution	chr7:g.55161606G>A	m-calpain		
EGFR	ENSG00000146648	Single base substitution	chr7:g.55174761G>A	m-calpain		
EGFR	ENSG00000146648	Single base substitution	chr7:g.55174764G>A	m-calpain		
EGFR	ENSG00000146648	Single base substitution	chr7:g.55191740C>T	m-calpain		
VWF	ENSG00000110799	Single base substitution	chr12:g.6110476C>T	m-calpain	ECM-receptor interaction, PI3K-Akt signaling pathway, Focal adhesion	https://www.kegg.jp/kegg-bin/search_pathway_text?map=hsa&keyword=VWF&mode=1&viewImage=true
VWF	ENSG00000110799	Single base substitution	chr12:g.5952401A>G	m-calpain		
VWF	ENSG00000110799	Single base substitution	chr12:g.6064369C>T	m-calpain		
VWF	ENSG00000110799	Single base substitution	chr12:g.6019244G>A	m-calpain		
VWF	ENSG00000110799	Single base substitution	chr12:g.5991920C>T	m-calpain		
ACAN	ENSG00000157766	Single base substitution	chr15:g.88840105C>T	m-calpain	Biosynthesis of nucleotide sugars	https://www.kegg.jp/kegg-bin/search_pathway_
ACAN	ENSG00000157766	Single base substitution	chr15:g.88871421G>A	m-calpain		
ACAN	ENSG00000157766	Single base substitution	chr15:g.88847327C>T	m-calpain		
PTK2	ENSG00000169398	Single base substitution	chr8:g.140879577G>A	m-calpain	Chemokine signaling pathway, Proteoglycans in cancer, Endocrine resistance, ErbB	https://www.kegg.jp/kegg-bin/search_pathway_text?map=hsa&keyw
PTK2	ENSG00000169398	Single base substitution	chr8:g.140789498G>A	m-calpain		
PTK2	ENSG00000169398	Single base substitution	chr8:g.140735384C>T	m-calpain		
PTK2	ENSG00000169398	Single base substitution	chr8:g.140746852G>A	m-calpain		
NFKB	ENSG00000100906	Single base substitution	chr14:g.35402053T>C	m-calpain		
SPTBN	ENSG00000115306	Single base substitution	chr2:g.54629655C>T	u-calpain	Nil	
SPTBN	ENSG00000115306	Single base substitution	chr2:g.54649849G>A	u-calpain		
SPTBN	ENSG00000115306	Single base substitution	chr2:g.54653775G>A	u-calpain		
SPTBN	ENSG00000115306	Single base substitution	chr2:g.54646228G>A	u-calpain		
SPTBN	ENSG00000115306	Single base substitution	chr2:g.54618166G>A	u-calpain		
SPTBN	ENSG00000115306	Single base substitution	chr2:g.54645346G>A	u-calpain		
SPTBN	ENSG00000115306	Single base substitution	chr2:g.54629050G>A	u-calpain		
SPTBN	ENSG00000115306	Single base substitution	chr2:g.54626083G>A	u-calpain		
SPTBN	ENSG00000115306	Single base substitution	chr2:g.54624906C>T	u-calpain		
SLC6A	ENSG00000142319	Single base substitution	chr5:g.1422013G>A	m-calpain	Amphetamine addiction, Parkinson disease, Cocaine	https://www.kegg.jp/kegg-bin/search_pathway_
SLC6A	ENSG00000142319	Single base substitution	chr5:g.1411313G>A	m-calpain		
SLC6A	ENSG00000142319	Single base substitution	chr5:g.1421959G>A	m-calpain		
GRM1	ENSG00000152822	Single base substitution	chr6:g.146398813G>A	u-calpain	Gap junction, Phospholipase D signaling pathway, FoxO signaling pathway	https://www.kegg.jp/kegg-bin/search_pathway_text?map=hsa&keyw
GRM1	ENSG00000152822	Single base substitution	chr6:g.146399402C>T	u-calpain		
GRM1	ENSG00000152822	Single base substitution	chr6:g.146159557C>T	u-calpain		

GRM1	ENSG00000152822	Single base substitution	chr6:g.146399089C>T	u-calpain	signaling pathway, Calcium signaling pathway	text?map=hsa&keyword=GRM1&mode=1&viewImage=true
GRM1	ENSG00000152822	Single base substitution	chr6:g.146399090G>A	u-calpain		
RB1	ENSG00000139687	Single base substitution	chr13:g.48380206C>T	u-calpain ;	Small cell lung cancer, Cell cycle, Pathways in cancer, Calcium	https://www.kegg.jp/kegg-bin/search_pathway_
RB1	ENSG00000139687	Single base substitution	chr13:g.48476806C>T	u-calpain ;		
RB1	ENSG00000139687	Single base substitution	chr13:g.48379613G>A	u-calpain ;		
ATP2E	ENSG00000157087	Single base substitution	chr3:g.10402243G>A	u-calpain	Calcium signaling pathway & others	https://www.kegg.jp/kegg-bin/search_pathway_text?map=hsa&keyword=ATP2B2&mode=1&viewImage=true
ATP2E	ENSG00000157087	Single base substitution	chr3:g.10449432G>A	u-calpain		
ATP2E	ENSG00000157087	Single base substitution	chr3:g.10402166C>T	u-calpain		
ATP2E	ENSG00000157087	Single base substitution	chr3:g.10342877G>A	u-calpain		
ATP2E	ENSG00000157087	Single base substitution	chr3:g.10449450G>A	u-calpain		
ATP2E	ENSG00000157087	Single base substitution	chr3:g.10360021G>A	u-calpain		
ATP2E	ENSG00000157087	Single base substitution	chr3:g.10375504C>T	u-calpain		
ATP2E	ENSG00000157087	Single base substitution	chr3:g.10375548G>A	u-calpain		
ATP2E	ENSG00000157087	Single base substitution	chr3:g.10359967G>A	u-calpain		
MBP	ENSG00000197971	Single base substitution	chr18:g.76984861C>T	m-calpain	Endocytosis, Human immunodeficiency virus 1 infection, Asthma	https://www.kegg.jp/kegg-bin/search_pathway_text?map=hsa&keyw
MBP	ENSG00000197971	Single base substitution	chr18:g.76980459T>C	m-calpain		
MBP	ENSG00000197971	Single base substitution	chr18:g.76984810C>T	m-calpain		
MBP	ENSG00000197971	Single base substitution	chr18:g.77016879G>A	m-calpain		
AMPD	ENSG00000116748	Single base substitution	chr1:g.114674758T>A	m-calpain	Nucleotide metabolism, Purine metabolism, Metabolic pathways	https://www.kegg.jp/kegg-bin/search_pathway_text?map=hsa&keyword=AMPD1&mode=1&viewImage=true
AMPD	ENSG00000116748	Single base substitution	chr1:g.114673683C>T	m-calpain		
AMPD	ENSG00000116748	Single base substitution	chr1:g.114684285G>A	m-calpain		
AMPD	ENSG00000116748	Single base substitution	chr1:g.114677399C>T	m-calpain		
AMPD	ENSG00000116748	Single base substitution	chr1:g.114673188G>A	m-calpain		
AMPD	ENSG00000116748	Single base substitution	chr1:g.114677466G>A	m-calpain		
AMPD	ENSG00000116748	Single base substitution	chr1:g.114674855G>A	m-calpain		
BID	ENSG00000015475	Single base substitution	chr22:g.17738226G>A	m-calpain ;	Apoptosis, p53 signaling pathway, Metabolic	https://www.kegg.jp/kegg-
BID	ENSG00000015475	Single base substitution	chr22:g.17739441C>T	m-calpain ;		
HTT	ENSG00000197386	Single base substitution	chr4:g.3206517G>A	u-calpain ;	Pathways of neurodegeneration - multiple diseases, Huntington disease	https://www.kegg.jp/kegg-bin/search_pathway_text?map=hsa&keyword=HTT&mode=1&viewImage=true
HTT	ENSG00000197386	Single base substitution	chr4:g.3174968G>A	u-calpain ;		
HTT	ENSG00000197386	Single base substitution	chr4:g.3199936C>T	u-calpain ;		
HTT	ENSG00000197386	Single base substitution	chr4:g.3208807C>T	u-calpain ;		
HTT	ENSG00000197386	Single base substitution	chr4:g.3236233G>A	u-calpain ;		
MET	ENSG00000105976	Single base substitution	chr7:g.116739963G>A	u-calpain	EGFR tyrosine kinase inhibitor resistance	https://www.kegg.jp/kegg-bin/search_pathway_
MET	ENSG00000105976	Single base substitution	chr7:g.116699722C>T	u-calpain		
MET	ENSG00000105976	Single base substitution	chr7:g.116699872C>T	u-calpain		
CPEB3	ENSG00000107864	Single base substitution	chr10:g.92144995C>T	m-calpain	Oocyte meiosis, Progesterone-mediated oocyte maturation	https://www.kegg.jp/kegg-bin/search_pathway_text?map=hsa&keyword=CPEB3&mode=1&viewImage=true
SPTB	ENSG00000070182	Single base substitution	chr14:g.64772770G>A	u-calpain	Pathways of neurodegeneration - multiple diseases,	https://www.kegg.jp/kegg-bin/search_pathway_
SPTB	ENSG00000070182	Single base substitution	chr14:g.64805010C>T	u-calpain		
SPTB	ENSG00000070182	Single base substitution	chr14:g.64795422C>T	u-calpain		
AMPD	ENSG00000133805	Single base substitution	chr11:g.10482141G>A	m-calpain	Nucleotide metabolism, Purine metabolism, Metabolic pathways	https://www.kegg.jp/kegg-bin/search_pathway_text?map=hsa&keyword=AMPD3&mode=1&viewImage=true
CASP9	ENSG00000132906	Single base substitution	chr1:g.15495294C>T	m-calpain	Pathways in cancer, Colorectal cancer, Non-	https://www.kegg.jp/kegg-
CASP9	ENSG00000132906	Single base substitution	chr1:g.15506990C>T	m-calpain		
TLN1	ENSG00000137076	Single base substitution	chr9:g.35707458G>A	m-calpain	Rap1 signaling pathway, Focal adhesion,	https://www.kegg.jp/kegg-
TLN1	ENSG00000137076	Single base substitution	chr9:g.35707796G>A	m-calpain		

ITGB7	ENSG00000139626	Single base substitution	chr12:g.53191896C>T	m-calpain	Cell adhesion molecules, Transcriptional misregulation in cancer, PI3K-Akt signaling pathway, Focal adhesion, ECM-receptor interaction, Regulation of actin cytoskeleton,	https://www.kegg.jp/kegg-bin/search_pathway_text?map=hsa&keyword=ITGB7&mode=1&viewImage=true
ACTN	ENSG00000072110	Single base substitution	chr14:g.68921009C>T	u-calpain ;	Adherens junction, Focal adhesion, Tight junction, Leukocyte transendothelial	https://www.kegg.jp/kegg-bin/search_pathway_text?map=hsa&keyw
ACTN	ENSG00000072110	Single base substitution	chr14:g.68909323G>A	u-calpain ;		
ACTN	ENSG00000072110	Single base substitution	chr14:g.68880030G>A	u-calpain ;		
ACTN	ENSG00000072110	Single base substitution	chr14:g.68885461C>T	u-calpain ;		
MIP	ENSG00000135517	Single base substitution	chr12:g.56453668C>T	m-calpain	Viral carcinogenesis, NF-kappa B signaling pathway, Signaling pathways regulating pluripotency of stem cells & others	https://www.kegg.jp/kegg-bin/search_pathway_text?map=hsa&keyword=MIP&mode=1&viewImage=true
EZR	ENSG00000092820	Single base substitution	chr6:g.158769858G>A	u-calpain	Leukocyte transendothelial	https://www.kegg.jp/kegg-
EZR	ENSG00000092820	Single base substitution	chr6:g.158771326G>A	u-calpain		
ANXA	ENSG00000135046	Single base substitution	chr9:g.73160363G>A	u-calpain	myotrophic lateral scleros	https://www.kegg.jp/kegg-
ANXA	ENSG00000135046	Single base substitution	chr9:g.73159368G>A	u-calpain		
ATP2E	ENSG00000067842	Single base substitution	chrX:g.153556136G>A	u-calpain	um signaling pathway & c	https://www.kegg.jp/kegg-bin/search_pathway_text?map=hsa&keyw
ATP2E	ENSG00000067842	Single base substitution	chrX:g.153549603G>A	u-calpain		
ATP2E	ENSG00000067842	Single base substitution	chrX:g.153553162C>T	u-calpain		
ATP2E	ENSG00000067842	Single base substitution	chrX:g.153553117G>A	u-calpain		
MYC	ENSG00000136997	Single base substitution	chr8:g.127738393C>T	calpain	Central carbon metabolism in cancer, Small cell lung cancer, Hepatocellular carcino, ma, Pathways in cancer, Transcriptional misregulation in cancer, Breast cancer, Colorectal cancer, Acute myeloid leukemia, Gastric cancer, Cellular senescence, MAPK signaling pathway, ErbB signaling pathway, Cell cycle, PI3K-Akt signaling pathway, Wnt signaling pathway, JAK-STAT signaling pathway, Proteoglycans in cancer, MicroRNAs in cancer, Chemical carcinogenesis - receptor activation, Endometrial, thyroid & bladder cancers, Chronic myeloid leukemia, p53 signaling pathway & others	https://www.kegg.jp/kegg-bin/search_pathway_text?map=hsa&keyword=MYC&mode=1&viewImage=true

PRKC	ENSG00000154229	Single base substitution	chr17:g.66803988C>T	u-calpain	MicroRNAs in cancer, Pathways in cancer, Proteoglycans in cancer, Glioma, MAPK signaling pathway, ErbB signaling pathway, Ras signaling pathway, Rap1 signaling pathway, Calcium signaling pathway, PI3K-Akt signaling pathway, Wnt signaling pathway, Focal adhesion, Gap junction, Leukocyte transendothelial migration, Chemical carcinogenesis - receptor activation, Non-small cell lung cancer, Hepatocellular carcinoma, Choline metabolism in cancer & others	https://www.kegg.jp/kegg-bin/search_pathway_text?map=hsa&keyword=PRKCA&mode=1&viewImage=true
MEF2I	ENSG00000116604	Single base substitution	chr1:g.156468159C>T	m-calpain	Apelin signaling pathway, cGMP-PKG	https://www.kegg.jp/kegg-
MEF2I	ENSG00000116604	Single base substitution	chr1:g.156468202G>A	m-calpain		
AMPH	ENSG00000078053	Single base substitution	chr7:g.38476954G>A	m-calpain	Fc gamma R-mediated phagocytosis & others	https://www.kegg.jp/kegg-bin/search_pathway_text?map=hsa&keyword=AMPH&mode=1&viewImage=true
AMPH	ENSG00000078053	Single base substitution	chr7:g.38462989G>A	m-calpain		
AMPH	ENSG00000078053	Single base substitution	chr7:g.38463004C>T	m-calpain		
AMPH	ENSG00000078053	Single base substitution	chr7:g.38476965C>T	m-calpain		
AMPH	ENSG00000078053	Single base substitution	chr7:g.38534963C>T	m-calpain		
CAPN	ENSG00000014216	Single base substitution	chr11:g.65209365G>A	u-calpain	Necroptosis, Apoptosis, Cellular senescence,	https://www.kegg.jp/kegg-bin/search_pathway_
CAPN	ENSG00000014216	Single base substitution	chr11:g.65188710C>T	u-calpain		
CAPN	ENSG00000014216	Single base substitution	chr11:g.65188022C>T	u-calpain		
IGFBP	ENSG00000115457	Single base substitution	chr2:g.216663964C>T	m-calpain	Nil	
GAD2	ENSG00000136750	Single base substitution	chr10:g.26219265C>A	calpain	Metabolic pathways & others	https://www.kegg.jp/kegg-bin/search_pathway_text?map=hsa&keyword=GAD2&mode=1&viewImage=true
RAD2	ENSG00000164754	Single base substitution	chr8:g.116847613C>T	u-calpain	Cell cycle	https://www.kegg.jp/kegg-bin/search_pathway_
RAD2	ENSG00000164754	Single base substitution	chr8:g.116863210C>T	u-calpain		
RAD2	ENSG00000164754	Single base substitution	chr8:g.116863243C>T	u-calpain		
SLC8A	ENSG00000100678	Single base substitution	chr14:g.70167009C>T	u-calpain ; m-calpain	Calcium signaling pathway & cadiomyopathy pathways	https://www.kegg.jp/kegg-bin/search_pathway_text?map=hsa&keyword=SLC8A3&mode=1&viewImage=true
SLC8A	ENSG00000100678	Single base substitution	chr14:g.70166786C>T	u-calpain ; m-calpain		
SLC8A	ENSG00000100678	Single base substitution	chr14:g.70167849C>T	u-calpain ; m-calpain		
SLC8A	ENSG00000100678	Single base substitution	chr14:g.70168140C>T	u-calpain ; m-calpain		
SLC8A	ENSG00000100678	Single base substitution	chr14:g.70048914C>T	u-calpain ; m-calpain		

Supplementary Document S1

1. **ITGB1 Wild-type**

KLLMIIHDRREFAKFEKEKMNAKWD**T**GENPIYKSAVTTVVNPKYEGK

2. **ITGB1 Mutant**

KLLMIIHDRREFAKFEKEKMNAKWD**M**GENPIYKSAVTTVVNPKYEGK

3. **ITGB3 Wild-type**

KLLITIHDRKEFAKFEEERARAKWDTANNPLYKEATSTFTNITYRGT

4. **ITGB3 Mutant (D749N)**

KLLITIHNRKEFAKFEEERARAKWDTANNPLYKEATSTFTNITYRGT

5. **ITGB3 Mutant (R786Q)**

KLLITIHDRKEFAKFEEERARAKWDTANNPLYKEATSTFTNITYQGT

6. **ITGB7 Wild-type**

RLSVEIYDRREYS**R**FEKEQQQLNWKQDSNPLYKSAITTTINPRFQEADSPTL

7. **ITGB7 Mutant (R760H)**

RLSVEIYDRREYS**H**FEKEQQQLNWKQDSNPLYKSAITTTINPRFQEADSPTL

Supplementary Document S2

```
#    -gapopen 10.0
#    -gapextend 0.5
#    -endopen 10.0
#    -endextend 0.5
#    -aformat3 pair
#    -sprotein1
#    -sprotein2
# Align_format: pair
# Report_file: stdout
########################################

#=======================================
#
# Aligned_sequences: 2
# 1: CAN2_HUMAN
# 2: CAN2_RAT
# Matrix: EBLOSUM62
# Gap_penalty: 10.0
# Extend_penalty: 0.5
#
# Length: 700
# Identity:     656/700 (93.7%)
# Similarity:   682/700 (97.4%)
# Gaps:           0/700 ( 0.0%)
# Score: 3530.0
#
#
#=======================================

CAN2_HUMAN         1 MAGIAAKLAKDREAAEGLGSHDRAIKYLNQDYEALRNECLEAGTLFQDPS     50
                     |||||.|||||||||||||||:|||||||||||.|||||||||.||||||
CAN2_RAT           1 MAGIAMKLAKDREAAEGLGSHERAIKYLNQDYETLRNECLEAGALFQDPS     50

CAN2_HUMAN        51 FPAIPSALGFKELGPYSSKTRGIEWKRPTEICADPQFIIGGATRTDICQG    100
                     |||:||:|||||||||||||||||||||||||||||||||||||||||||
CAN2_RAT          51 FPALPSSLGFKELGPYSSKTRGIEWKRPTEICADPQFIIGGATRTDICQG    100

CAN2_HUMAN       101 ALGDCWLLAAIASLTLNEEILARVVPLNQSFQENYAGIFHFQFWQYGEWV    150
                     |||||||||||||||||||||||||||:||||||||||||||||||||||
CAN2_RAT         101 ALGDCWLLAAIASLTLNEEILARVVPLDQSFQENYAGIFHFQFWQYGEWV    150

CAN2_HUMAN       151 EVVVDDRLPTKDGELLFVHSAEGSEFWSALLEKAYAKINGCYEALSGGAT    200
                     ||||||||||||||||||||||||||||||||||||||||||||||||||
CAN2_RAT         151 EVVVDDRLPTKDGELLFVHSAEGSEFWSALLEKAYAKINGCYEALSGGAT    200

CAN2_HUMAN       201 TEGFEDFTGGIAEWYELKKPPPNLFKIIQKALQKGSLLGCSIDITSAADS    250
                     |||||||||||||||||:||||||||||||||:|||||||||||||||||
CAN2_RAT         201 TEGFEDFTGGIAEWYELRKPPPNLFKIIQKALEKGSLLGCSIDITSAADS    250
```

Supplementary Document S2

```
CAN2_HUMAN       251 EAITFQKLVKGHAYSVTGAEEVESNGSLQKLIRIRNPWGEVEWTGRWNDN     300
                     ||:|:|||||||||||||||||||:||||||||||||||:|||||:||||
CAN2_RAT         251 EAVTYQKLVKGHAYSVTGAEEVESSGSLQKLIRIRNPWGQVEWTGKWNDN     300

CAN2_HUMAN       301 CPSWNTIDPEERERLTRRHEDGEFWMSFSDFLRHYSRLEICNLTPDTLTS     350
                     ||||||:|||.|..||.|.||||||||||||||||||||||||||||||.
CAN2_RAT         301 CPSWNTVDPEVRANLTERQEDGEFWMSFSDFLRHYSRLEICNLTPDTLTC     350

CAN2_HUMAN       351 DTYKKWKLTKMDGNWRRGSTAGGCRNYPNTFWMNPQYLIKLEEEDEDEED     400
                     |:|||||||||||||||||||||||||||||||||||||||||||||:||
CAN2_RAT         351 DSYKKWKLTKMDGNWRRGSTAGGCRNYPNTFWMNPQYLIKLEEEDEDDED     400

CAN2_HUMAN       401 GESGCTFLVGLIQKHRRRQRKMGEDMHTIGFGIYEVPEELSGQTNIHLSK     450
                     ||.|||||||||||||||||||||||||||||||||||||:|||||||||
CAN2_RAT         401 GERGCTFLVGLIQKHRRRQRKMGEDMHTIGFGIYEVPEELTGQTNIHLSK     450

CAN2_HUMAN       451 NFFLTNRARERSDTFINLREVLNRFKLPPGEYILVPSTFEPNKDGDFCIR     500
                     |||||.||||||||||||||||||||||||||:||||||||:|:||||||
CAN2_RAT         451 NFFLTTRARERSDTFINLREVLNRFKLPPGEYVLVPSTFEPHKNGDFCIR     500

CAN2_HUMAN       501 VFSEKKADYQAVDDEIEANLEEFDISEDDIDDGFRRLFAQLAGEDAEISA     550
                     ||||||||||.||||||||:||.:.:|:||.|||||||||||||||||||
CAN2_RAT         501 VFSEKKADYQTVDDEIEANIEEIEANEEDIGDGFRRLFAQLAGEDAEISA     550

CAN2_HUMAN       551 FELQTILRRVLAKRQDIKSDGFSIETCKIMVDMLDSDGSGKLGLKEFYIL     600
                     ||||||||||||||:||||||||||||||||||||.||||||||||||||
CAN2_RAT         551 FELQTILRRVLAKREDIKSDGFSIETCKIMVDMLDEDGSGKLGLKEFYIL     600

CAN2_HUMAN       601 WTKIQKYQKIYREIDVDRSGTMNSYEMRKALEEAGFKMPCQLHQVIVARF     650
                     |||||||||||||||||||||||||||||||||||||:||||||||||||
CAN2_RAT         601 WTKIQKYQKIYREIDVDRSGTMNSYEMRKALEEAGFKLPCQLHQVIVARF     650

CAN2_HUMAN       651 ADDQLIIDFDNFVRCLVRLETLFKIFKQLDPENTGTIELDLISWLCFSVL     700
                     |||:||||||||||||||||.||||||||||||||||:|||||||.||||
CAN2_RAT         651 ADDELIIDFDNFVRCLVRLEILFKIFKQLDPENTGTIQLDLISWLSFSVL     700
```

Supplementary Figure S1

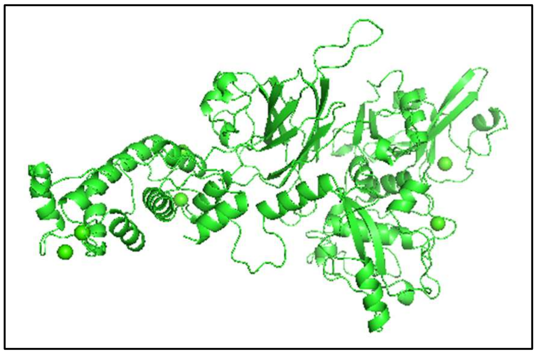

(a)

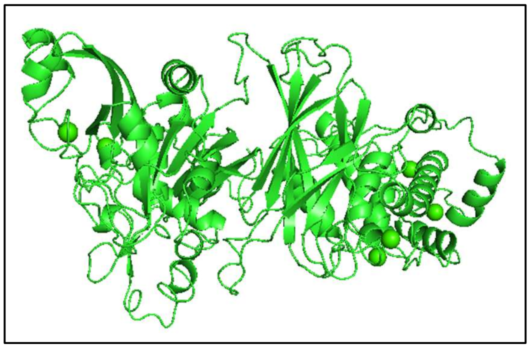

(b)

Figure: **(a)** Modelled and simulated structure of calpain-1; **(b)** Modelled and simulated structure of calpain-2

Supplementary Figure S2

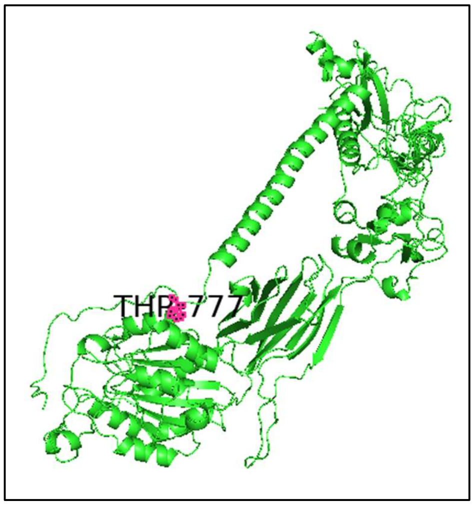

(a)

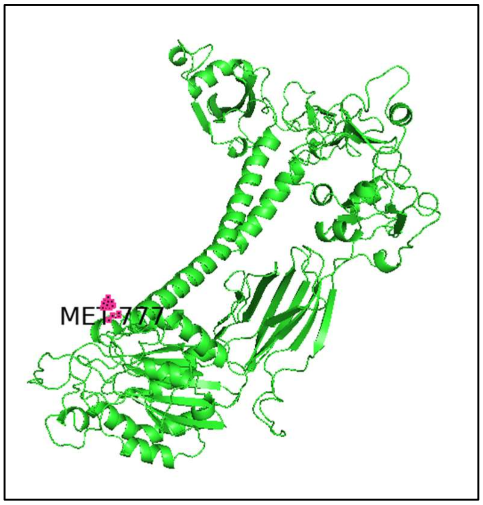

(b)

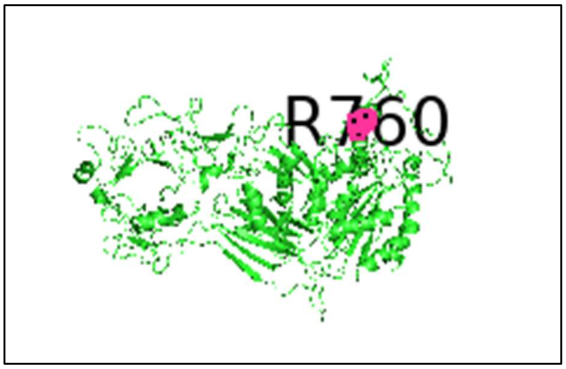

(c)

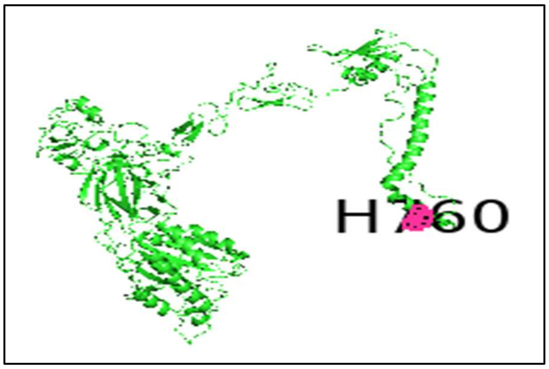

(d)

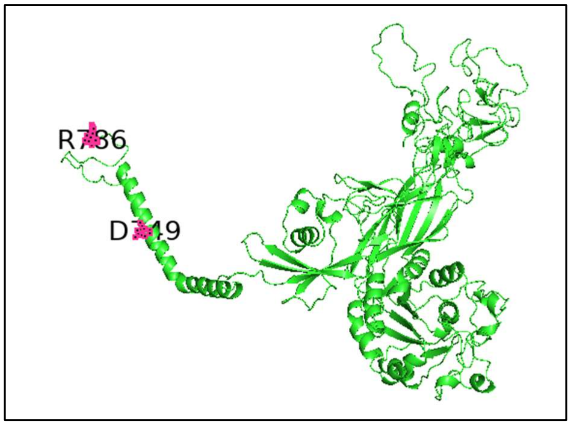

(e)

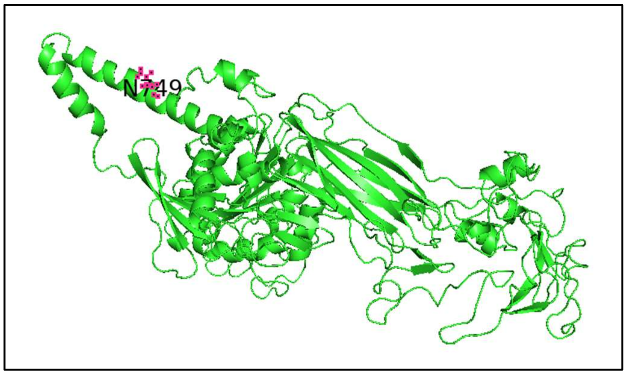

(f)

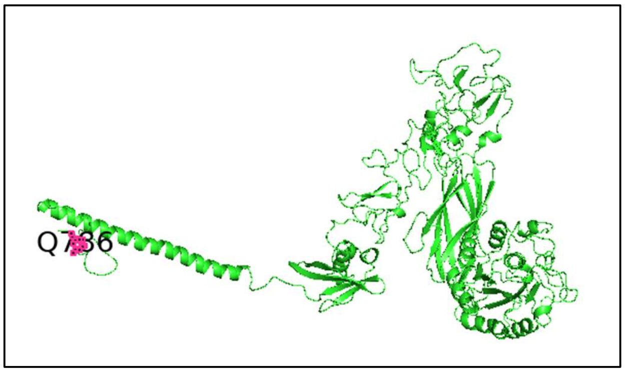

(g)

Figure: **(a, b)** Modelled and simulated structures of wild-type and mutant ITGB1 respectively, **(c, d)** Modelled and simulated structures of wild-type and mutant ITGB7 respectively and **(e, f, g)**: Modelled and simulated structures of wild-type and mutant ITGB3 (D749N & R786Q) respectively.
